# Calmodulin-specific small interfering RNA induces consistent expression suppression and morphological changes in *Echinococcus granulosus*

**DOI:** 10.1038/s41598-019-40656-w

**Published:** 2019-03-07

**Authors:** Seyed Mohammad Mousavi, Ali Afgar, Mohammad Ali Mohammadi, Seifollah Mortezaei, Balal Sadeghi, Majid Fasihi Harandi

**Affiliations:** 1 Research Center for Hydatid Disease in Iran, Kerman University of Medical Sciences, Kerman, 7616914115 Iran; 2 Shahid Bahonar University of Kerman, Faculty of Veterinary Medicine, Department of Food Hygiene and Public Health, Kerman, Iran

## Abstract

Among parasitic helminths, biological features of *Echinococcus granulosus* have been a focus of particular interest in biology and medicine. The determinants and underlying molecular mechanisms of *Echinococcus* development in different host settings is largely unknown. The phenomenal bi-directional development of *E. granulosus* protoscoleces into multi-proglottid and/or microcysts, is a fascinating feature of the parasite cultivation. Calmodulin (CaM) is the major intracellular Ca2+ binding protein in plant and animal organisms. Many Ca^2+^-related processes in the physiology of eukaryotic organisms are CaM-dependent, however little is known on the role of CaM in platyhelminths growth and development. Small interfering (si) RNA-induced manipulations of the genes involving in the parasite development is an opportunity to explore novel approaches for cystic echinococcosis (CE) prevention and management. Regarding the fundamental role of CaM in cellular function of the parasites, in this study, we investigated the molecular and morphological changes induced by siRNA on CaM in different *in vitro* stages of *E. granulosus*. Three developmental stages of the tapeworm, protoscoleces, microcysts and strobilated worms, were cultivated *in vitro* in mono- and di-phasic media and three delivery methods, i.e. electroporation, soaking and electro-soaking, were used for RNA interference. The level of mRNA suppression as well as the phenotypic changes of the parasites were measured. Following RNA interference, EgCaM mRNA suppressions of 65–99% were recorded in different stages of the tapeworm as compared to untreated/unrelated siRNA controls. Lower viability, growth retardation, morphological abnormalities as well as EgCaM expression suppression were documented in the parasite implying potential of siRNA technology for the prevention and management of CE.

## Introduction

*E. granulosus* is a zoonotic platyhelminth causing cystic echinococcosis (CE) in human and livestock. The life cycle of *E. granulosus* is complex and required two mammalian hosts, leading to many morphological, biochemical, and physiological alterations in the parasite. *Echinococcus* metacestode called hydatid cyst, develops in the viscera of herbivorous intermediate hosts (livestock and humans) and can infect carnivorous final host (most commonly dogs) via feeding with infected organs. This parasite has a worldwide distribution and it has been estimated to infect 2–3 million people across the globe^1^.

The gravid worm shed eggs in dog small intestine which are disseminated in the environment via defecation. Infection of the intermediate host occurred through ingestion of infective eggs, followed by the release of oncospheres in the small intestine. The oncosphere subsequently migrates via the portal system to various organs and tissues, mainly liver and lungs, where it develops into a unilocular, fluid-filled cyst comprised of an inner germinal layer which responsible for production of the infective stage, a structure called protoscolex (PSC), an extracellular matrix in the middle called laminated layer that is unique to the genus *Echinococcus*, and an external host-derived adventitial layer formed in response to the parasite^2^. As a model organism *Echinococcus granulosus* has been demonstrated as a valuable biological entity influencing advancements in developmental biology of flatworms^3^.

Following *Echinococcus* cultivation in appropriate diphasic and/or monophasic media the phenomenon of bi-directional development of *E. granulosus* protoscoleces into multi-proglottid and/or microcysts, is a fascinating feature of *Echinococcus* biology. This provides us an opportunity to study genes involving in the parasite development and to manipulate the parasite for disease management and prevention^4–6^. Understanding the determinants and molecular mechanisms in the parasite development in different host settings offers suitable tools for prevention and control of CE^2^.

Calmodulin (CaM), is a multifunctional intermediate calcium sensor protein expressed in all eukaryotic organisms. The functions of CaM include Ca2+ binding and alteration of calcium signal transduction pathway to regulate a multitude of biological processes, such as cytoskeletal assembly/reorganization, activation of phosphorylase kinase, abiotic stress responses, neurotransmission, smooth muscle contraction, metabolism and cell motility^7,8^. Wang *et al*. have used Immunohistochemical localizations on different stages of the parasite, suggesting the expression of *E. granulosus* CaM (EgCaM) in the tegument tissues, protoscoleces parenchymal region, germinal layer as well as in the adult stage. The study showed physiologically active regions for EgCaM expression and suggested the crucial role of Ca2+ signaling pathway in the growth and development of the helminth and its function between *E. granulosus* and its host^9^.

In recent decade, there has been increasing interest in RNA interference (RNAi) technology in the field of biology and development. Our understanding of the genetic basis of development in invertebrates is limited. Small interfering RNAs (siRNAs) provides us a valuable tool to improve our knowledge on the developmental biology of Platyhelminthes^10^. Molecular methods based on RNAi or Post-Transcriptional Gene Silencing (PTGS) are the new millennium tools that provide information on gene function and characterization of the genes involving in regeneration and development. RNA interference mediated by siRNA with perfect homology to their target can cause silencing of specific genes for experimental and therapeutic purposes^11,12^.

RNAi has been successfully applied to numerous helminth organisms to date. Silencing of the gene encoding *Fasciola hepatica* leucine aminopeptidase (LAP) with siRNAs as well as double-stranded RNAs (dsRNA), suppressed mRNA and protein expression for up to 3 weeks in the newly excysted juveniles^13^. RNAi has been used to suppress a number of *E. multilocularis* endogenous genes expression in protoscoleces^14^. Protoscoleces electroporation of genes encoding the 14–3–3 and *elp* with siRNA resulted in reduced expression of up to 21.8 and 35.5% of the genes respectively, compared to untreated control. In addition, the target proteins have been significantly reduced on day 15^14^.

CaM-specific RNAi technology has been utilized to determine the functional importance of calmodulin in several helminth parasites, however there are very few data on the role of CaM function in *E. granulosus*. There are reports for schistosomes^15,16^, the liver flukes *Fasciola gigantica* and *F. hepatica*^17,18^ and *Caenorhabditis elegans*^19^. Suppression of calmodulin mRNA by feeding *Schistosoma mansoni* with dsRNA resulted in a phenotype characterized by waves of contraction in adult worms but not in schistosomula^16^. RNAi has also been used to determine the biological function of *F. hepatica* calmodulin *in vitro*. Stunted growth and lower viability were documented after treating the *in vitro* cultured flukes with dsRNAs and siRNA^20^. The purpose of the present study was to determine the effect of RNA interference on EgCaM expression in different developmental stages of *E. granulosus in vitro*.

## Results

### *In vitro* cultivation of protoscoleces

**Figure 1:**
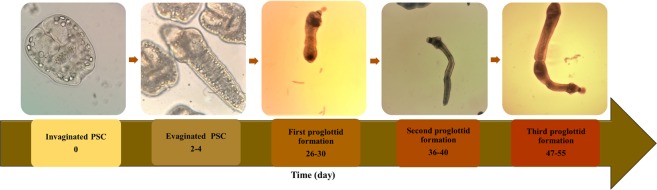
Timeline (days) of *in vitro* developmental stages of *Echinococcus granulosus* cultured in CMRL 1066 medium for 55 days.

The parasite was characterized using PCR-sequencing and identified as the G1 genotype of *E. granulosus* (Accession Number MG832791). *E. granulosus* protoscoleces were successfully cultivated in both monophasic and diphasic media. The protoscoleces started to develop proglottization in diphasic culture and the first proglottids were observed on day 28. However, the full grown three-proglottid strobilated forms were developed after 55 days of cultivation. Egg production in the terminal proglottid was not observed under the culture conditions (Fig. 1). In monophasic condition the protoscoleces were developed into conspicuous microcysts after 45 days. The culture was characterized by wide proliferation of parasite vesicles with no development of new protoscoleces.

### The effects of suppressed calmodulin on the protoscoleces

**Figure 2:**
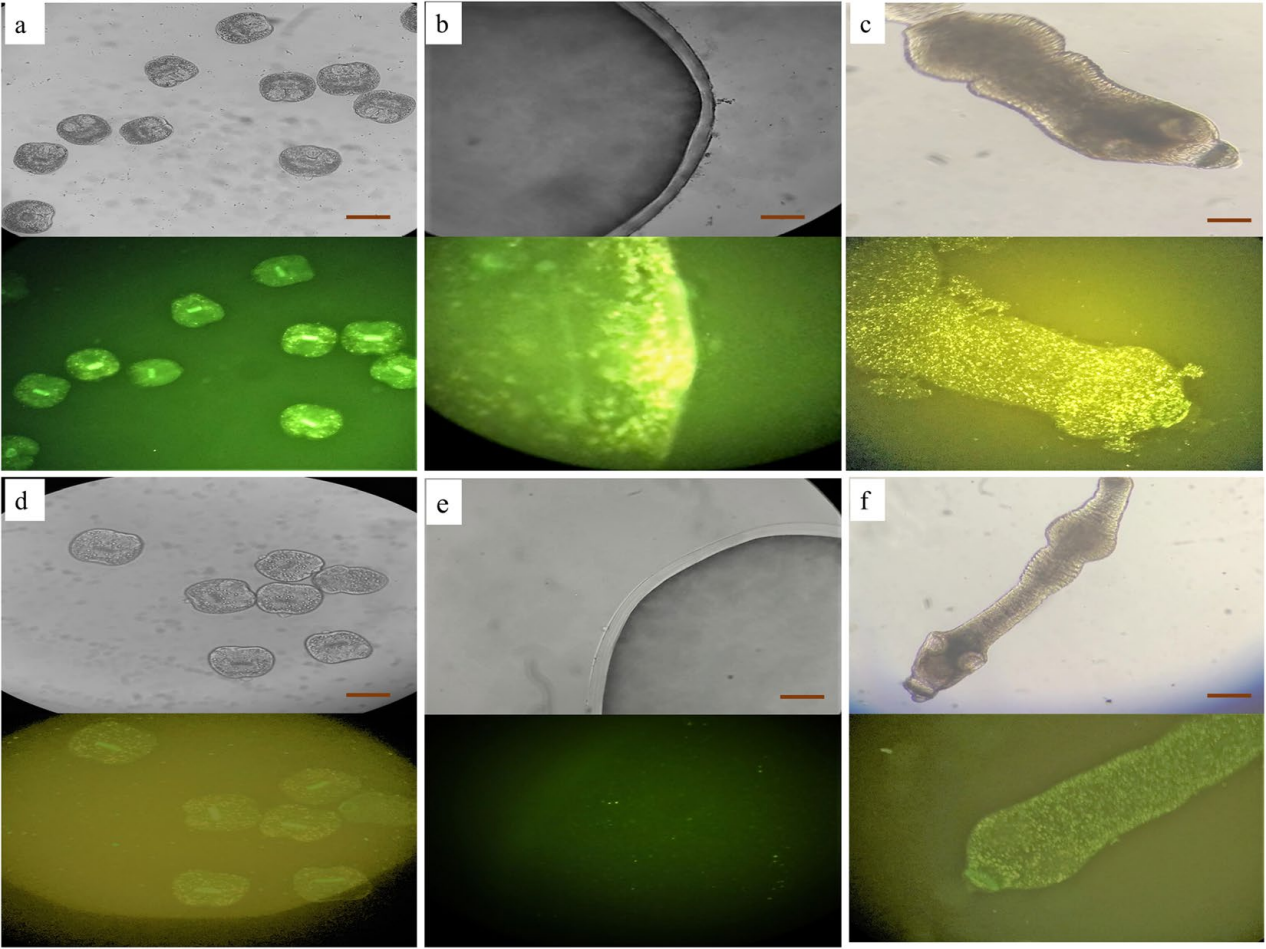
Representative images of different *in vitro* stages of *Echinococcus granulosus* showing siRNA uptake of the parasites under light and fluorescent microscopy. (**a**–**c**) siRNA uptake by protoscoleces (**a**), microcyst (**b**) and strobilated worm (**c**) in comparison to the corresponding Negative Control siRNA images (**d**–**f**). Scale bar = 200 μm.

Previous reports had demonstrated the activity of experimental RNAi in protoscoleces, either by electroporation or soaking^14,21^. Initially, we focused on uptake of fluorescently labeled siRNA in protoscoleces by fluorescent microscope. Fluorescently labeled siRNA was detected in treated parasites comparing to no or very low level of autofluorescence in untreated parasites (Fig. 2a).

**Figure 3:**
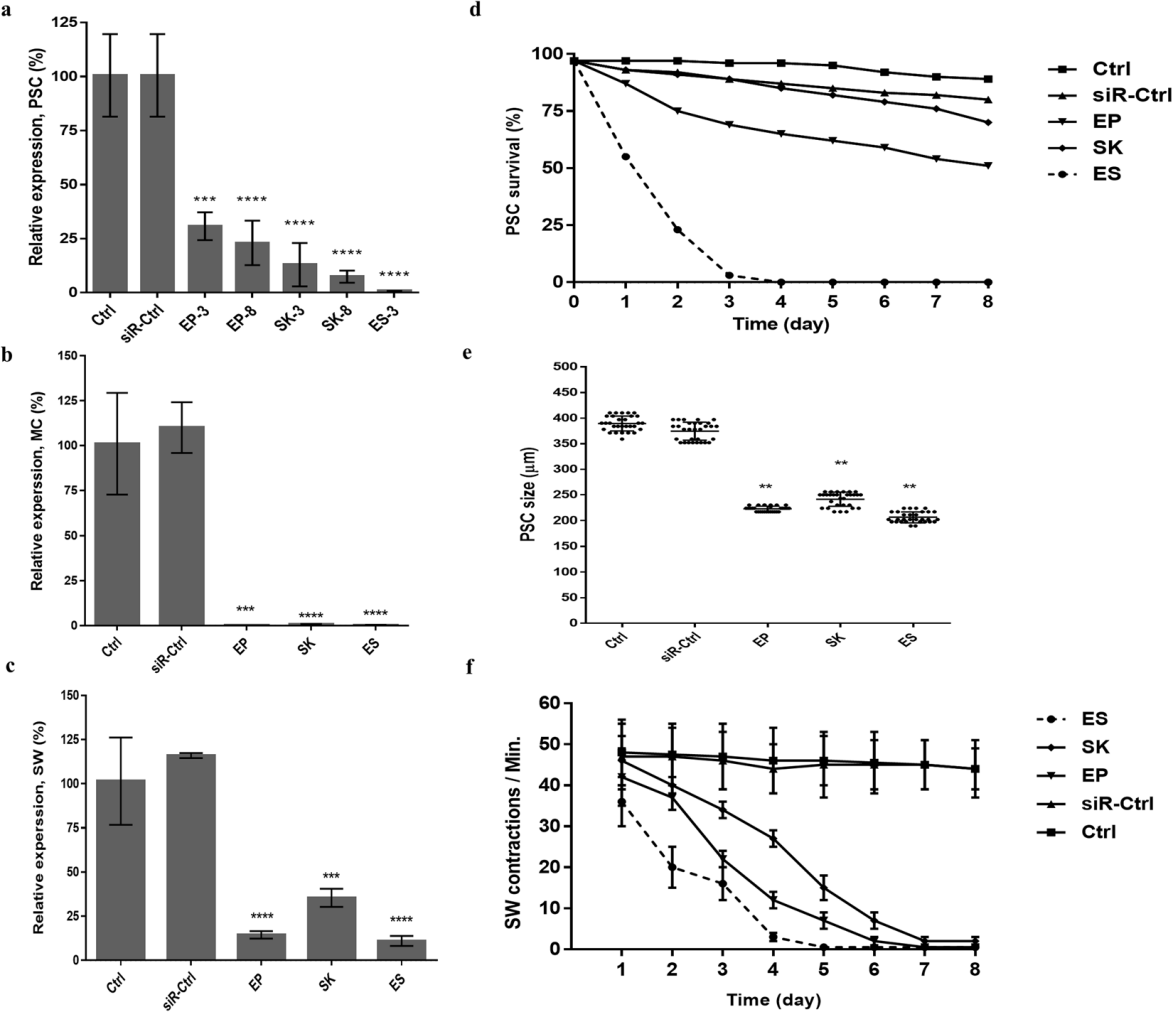
Molecular (**a**–**c**) and biological (**d**–**f**) changes induced by *Echinococcus granulosus* Calmodulin (EgCaM)-specific siRNA using three delivery methods in different developmental stages *in vitro*. (**a**–**c**) EgCaM expression profile in different *in vitro* stages: protoscoleces (PSC) 3 and 8 days after electroporation (EP), soaking (SK) and electro-soaking (ES); microcysts (MC) and strobilated worms (SW). (**d**) Viability changes of protoscoleces treated with EgCaM-specific siRNA. (**e**) Size changes of 30 protoscoleces treated with EgCaM-specific siRNA. (**f**) Changes in body contractions per minute in the strobilated worms treated with EgCaM-specific siRNA. Data was compared to the controls: control protoscoleces (Ctrl) and negative siRNA control (siR-Ctrl). The gene expression data for siR-Ctrl were only demonstrated for electro-soaking method. Bars show the mean ± standard deviation (SD) derived from duplicates experiments. (**P < 0.01, ***P < 0.001, ****P < 0.0001).

Following RNA interference, 69 to 99% EgCaM mRNA suppression was recorded as compared to untreated controls (Fig. 3a). We found that electro-soaking method exerted the strongest transcript level suppression among three delivery methods (Table 1). Hence the gene expression data for negative control siRNA (siR-Ctrl) were only demonstrated for electro-soaking method. There was no significant difference in gene silencing between the negative control siRNA and no-treatment control groups.

**Table 1:** Expression suppression induced by *Echinococcus granulosus* Calmodulin (EgCaM)-specific siRNA in different developmental stages of *Echinococcus granulosus* using three delivery methods.

Stage (Day)	Expression Suppression (%)		
	Electroporation	Soaking	Electro-Soaking
Protoscoleces (3)	69	77	99
Protoscoleces (8)	87	92	—
Microcysts (8)	99	99	99
Strobilated worms (8)	86	65	89

The effect of EgCaM suppression on viability and size changes in protoscoleces was evaluated after siRNA treatment. Our results indicated the remarkable effect of EgCaM suppression on the viability of protoscoleces on day 8 post treatment. As shown in Fig. 3d, the viability of the parasites was decreased to 3–49% depending on different delivery methods. The protoscoleces were most affected by RNA interference using electro-soaking so that no protoscoleces were viable after three days post treatment.

Morphometric data are indicative of growth inhibition in siRNA-treated protoscoleces. A significant growth retardation was observed after day 8 in the protoscoleces exposed to siRNA compared to the control parasites (Fig. 3e). The protoscoleces size in the control groups were increased towards microcyst development (mean size of protoscoleces: control = 389 ± 14.5 μM; negative siRNA = 375 ± 17.4 μM) while the growth and development of siRNA-treated parasites were inhibited (mean size = 224 ± 10.07 μM).

### The effects of suppressed calmodulin on the microcyst

Figure 2b shows the fluorescence produced by siRNA incorporation into the microcyst wall. Relative expression of EgCaM eight days after siRNA delivery was reduced by 99% (Fig. 3b, Table 1).

**Figure 4:**
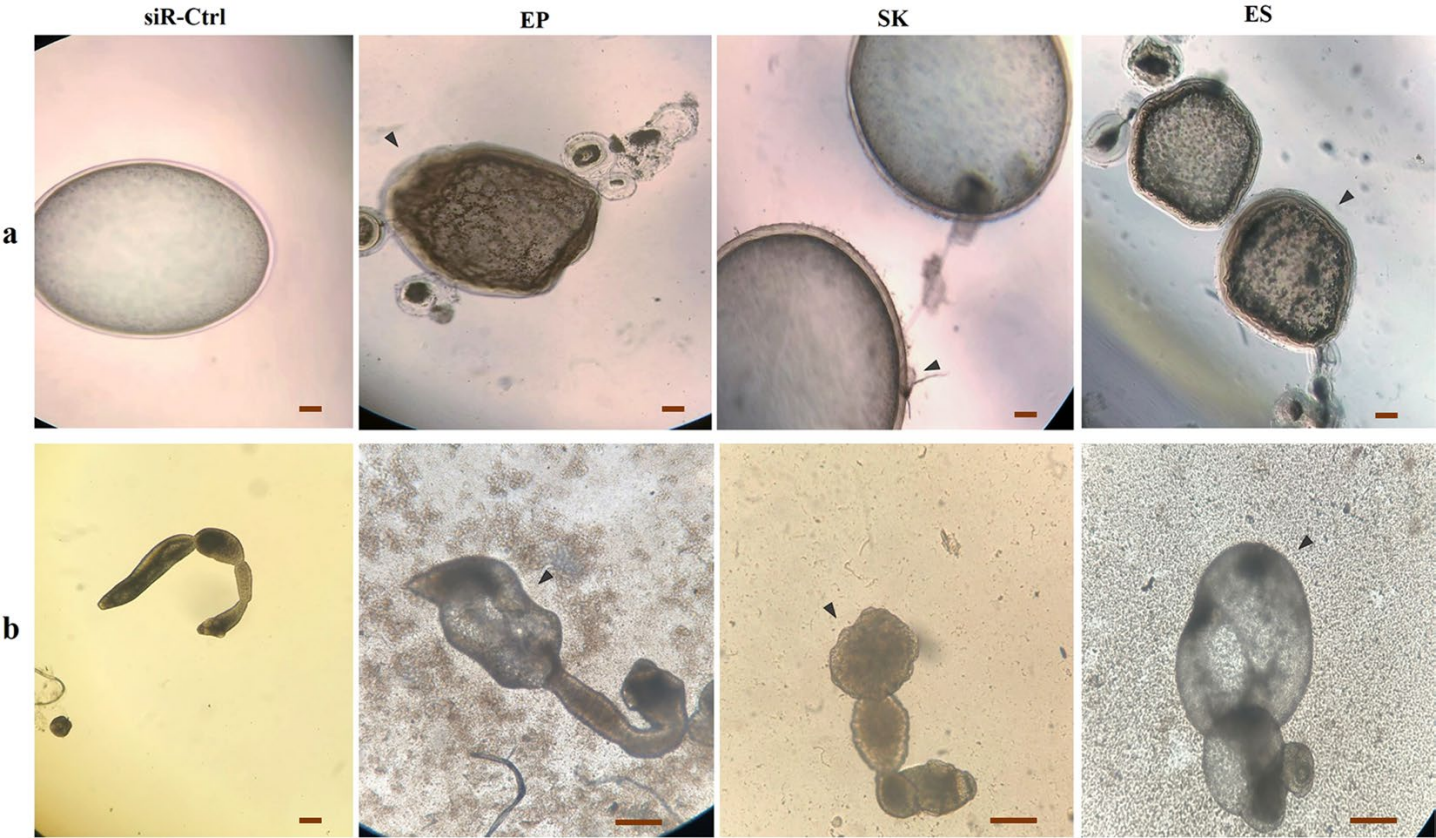
Morphological changes in *Echinococcus granulosus* treated with Calmodulin (EgCaM)-specific siRNA *in vitro*. Effect of EgCaM suppression on microcysts (**a**) and strobilated worms (**b**) using three delivery methods, electroporation (EP), soaking (SK), electro-soaking (ES) compared to the negative siRNA control (siR-Ctrl). Note the morphological changes (arrowheads) in the microcysts (outer layer irregularities and shrinkage, malformations and darkened edges) and the strobilated worms (morphological abnormalities and body swelling) compared to the controls. Scale bar = 200 μm.

Some morphological changes were noticed in siRNA-treated microcysts compared to the control groups. As shown in Fig. 4a the treated microcysts were noticeably darker with irregular outer layer compared to the controls with transparent microcysts and smooth surface. The electro-soaked microcysts were the most affected group when compared with microcysts treated by soaking or electroporation.

### The effects of suppressed calmodulin on the strobilated forms

There was successful uptake of fluorescently labeled target siRNA by strobilated forms cultured *in vitro*. This was visible throughout the helminth body. In comparison to the metacestode stages we observed high levels of autofluorescence in the strobilated control groups (no-treatment and unrelated negative siRNA controls), however the fluorescence pattern was distinct and readily distinguishable in the parasites tegument treated with siRNA. (Fig. 2c).

siRNA treatment of the strobilated forms induced a range of 65 to 89% suppression of mRNA for EgCaM as assessed after 8 days post treatment (Fig. 3c, Table 1). As shown in Fig. 4b EgCaM silencing led to severe morphological abnormalities and reduced activity compared to the both controls. Seven days after EgCaM silencing the helminths were severely damaged after electroporation and electro-soaking.

As shown in Fig. 3f significant decrease in body contractions in the strobilated worms was noted, indicating the contribution of EgCaM silencing by siRNA. In the electro-soaking group while on day 2, on average 20 contractions per minute were recorded, on day 5 no body contractions were found, compared to the untreated control and negative control siRNA with an average of 47 and 46 contractions per minute respectively.

## Discussion

RNA-dependent gene silencing has been increasingly utilized as a reverse-genetics approach in a variety of helminth organisms to generate functional gene knockdowns^22^. RNAi provides us with a powerful tool for predicting potential roles of genes involved in host-parasite interactions and vital processes essential for survival in several helminths such as *Brugia malayi*^23–25^ *Ascaris suum*^26,27^ *C. elegans*^19,28^, *Schistosoma* species^29–31^ and *F. hepatica*^13,32^. Although RNAi has been widely used for silencing genes in nematodes and trematodes, there are few studies applying RNAi on cestode parasites e.g. adult *Moniezia expansa*^33^, and protoscoleces of *E. multilocularis*^14^ and *E. granulosus*^21^. In the present study RNA interference technology was applied to suppress CaM in *E. granulosus*. Although CaM has been extensively studied and well characterized in several parasitic and free-living helminths^7,16,19^, there are very few data on the function and biological properties of CaM in *E. granulosus*. In the current investigation, we used siRNA sequences for EgCaM suppression to observe effects on different developmental stages of *E. granulosus in vitro*. All developmental stages of *E. granulosus* delivered with EgCaM-specific siRNA showed a robust suppression in EgCaM gene expression leading to obvious phenotypic changes (Figs 3 and 4). These results suggested a probable role for EgCaM in the growth and development of*E. granulosus*.

Suppression in CaM gene expression by siRNA and dsRNA has been investigated in a number of platyhelminthes including *S. mansoni*^16^, *F. hepatica*^7,20^ as well as the free-living nematode, *C. elegans*^19^. The results indicate CaM, as a transporter of Ca^2+^, plays an important role in the helminths growth, motility and metabolism.

Using different siRNA delivery methods produced a consistent suppression at mRNA level. The strongest effect (at least 92% suppression) was observed following electro-soaking in all developmental stages of *E. granulosus* (Table 1). It has been shown that electroporation produces pores in some biological membranes, favoring entrance of siRNA in the transfection buffer^13^.

We observed a robust suppression in EgCaM gene expression, resulted in phenotypic changes and viability and growth inhibition in siRNA-treated protoscoleces (Fig. 3). Mizukami *et al*. successfully suppressed 14-3-3 and *elp* genes expression by 21.8% and 35.5% respectively. Also, in the analysis of viability, 14-3-3, and *elp* siRNA-treated samples displayed 58.0 ± 23.0, and 55.1 ± 14.6% viability on day 15, respectively, compared to the untreated control^14^. This is in line with our data on the viability of protoscoleces after siRNA-mediated CaM suppression (Fig. 3d).

Our findings indicate that EgCaM specific siRNA in the protoscoleces induces growth retardation and inhibits protoscoleces transformation to microcysts compared to the controls (Fig. 3e). This imply that the reduction in CaM expression may prevent growth and development processes as shown in previous studies that linked CaM with the growth and development of other helminth species including miracidial transformation to sporocysts in *S. mansoni*^15^ and reduced growth in the free-living nematode *C. elegans*^19^ and the liver fluke *Fasciola hepatica*^20^. The growth retardation induced by RNAi has been demonstrated also in several other genes in *S. manson*^34–36^. Tegument malformations have been documented after using Eg-TSP1-specific siRNA on the soaked protoscoleces of *E. granulosus*^21^.

In the current study RNAi-based interventions were associated with strong suppression of EgCaM in siRNA-treated microcysts, causing shrinkage and darkness in the outer wall of the microcysts (Figs 3b and 4a). Wang *et al*. showed EgCaM expression in the germinal layer and speculated that an unknown calcium-dependent mechanism occurs in this layer^9^. More in-depth investigations are needed on the probable role of CaM in the stability of the microcyst wall and germinal layer.

After 8 days of siRNA treatment on the strobilated forms, 65–89% EgCaM expression suppression was observed (Table 1). In a recent study conducted on *S. mansoni* by Guidi *et al*., Sm-CaM suppressions ranging from 70 to 95% were documented using dsRNA. Waves of contraction/dilation in the adult worms were also reported in the treated worms^16^. Another study showed siRNA-mediated knockdown of a 24 kDa calcium-regulated heat-stable protein (CRHSP-24) in juvenile *S. japonicum*, inducing death or changes in the parasite morphology^37^.

In the strobilated worms we noted significant phenotype changes including severe morphological abnormalities and reduced body contractions from day 2 onwards (Fig. 4b). It seems the suppression of EgCaM in the strobilated forms of *E. granulosus* leads to decreased motility and strobilar contractions (Fig. 3f). While parasites in the untreated control and negative siRNA groups were quite active with normal contractions, no body contractions were found in their siRNA-treated counterparts. The association of motility and CaM expression is well-documented in the literature. In 1990s, Ashizawa *et al*. were first to demonstrate disturbed motility of spermatozoa after experimental disruption of CaM function^38^. In general, Ca^2+^-dependent protein kinases like Calmodulin, MAPK and Polo-Like Kinase (PLK) are proved to play a key role in the motility-related functions in invertebrates^16,39,40^. However, using both RNAi and the CaM inhibitor, Trifluoperazine, McCammick *et al*. showed a significant increase in motility, migration and movement of the juvenile *F. hepatica*^20^.

Generalizations of *in vitro* data to *in vivo* experiments is difficult. Therefore, cautions have to be made in the interpretation of culture data at the host level^41^. As *in vitro*-reared *E. granulosus* do not develop as much as they do in the hosts, we could not precisely predict the behavior of the helminth in its natural habitat in the host^42^. As the dsRNAs are generally induced more persistent effects on target genes, this study would be supplemented by using an EgCaM-specific dsRNA. However, using dsRNAs has its own limitations and challenges^43,44^. Another prospect for RNAi-induced gene silencing in *E. granulosus* is to investigate the interference impacts at protein level using western blotting and other protein-based tools as well as further in-depth *in vivo* studies followed by deep sequencing analyses.

The present study used siRNA for suppressing EgCaM in different developmental stages of *E. granulosus*. The results indicate the significant effect of siRNA treatments on the phenotype as well as the gene expressions in all development stages of *E. granulosus* cultured in mono- and di-phasic media using different delivery methods. The study demonstrated that EgCaM is essential for viability, growth and development of the protoscoleces. Different phenotypic changes and transcript knockdowns observed in the present study encourage further investigations towards the development of novel therapeutic agents against cystic echinococcosis.

## Methods

### *In vitro* cultivation of protoscoleces

Parasite specimens were obtained by dissection of livers from naturally infected sheep at the municipal abattoir of Kerman, southeastern Iran with consent from animal handlers and the abattoir veterinary officer. Animals were slaughtering as part of the normal daily practice in the abattoir. The study was approved by the University Ethics Review Committee (code 95000288). All procedures were performed according to the University guidelines and regulations. The infected organs were immediately transferred to the Helminthology Lab, Dept of Medical Parasitology, Kerman University of Medical Sciences. After careful examinations of fertility and viability, one single viable cyst was finally selected for further experiments. Genotyping has been carried out using mitochondrial cox1 PCR-sequencing and the sequence was submitted to GenBank. Protoscoleces used for *in vitro* cultivation, were obtained from a single sheep liver cyst. Therefore, the microcysts and strobilated worms were derived from the cultivation of protoscoleces obtained from the same cyst.

Under sterile conditions the hydatid fluid containing protoscoleces was aspirated with a 50 ml syringe and then the laminated/germinal layer were removed. The aspirate as well as the cyst layers were carefully washed five times with PBS containing 100 U/ml penicillin and 100 μg/ml streptomycin (PBS-PS). The number of protoscoleces per ml was adjusted to 1 × 10^4^ protoscoleces in 0.9% NaCl solution with a viability rate of at least 95%. Before cultivation the viability of the protoscoleces was checked by 0.1% aqueous eosin under a light microscope^44,45^. Two layers of sterile gauze were used to release protoscoleces from the brood capsules.

The protoscoleces were used for *in vitro* culture in diphasic and monophasic media to reach the strobilated as well as the microcyst forms, respectively, according to the method described by Smyth *et al*.^5,45^. The diphasic medium was S.10E.H, consisted of two phases: (i) the liquid phase, containing 260 mL of CMRL 1066 medium (Gibco, Grand Island, NY,), 100 mL of heat-inactivated fetal calf serum (FCS, Gibco-BRL, Gaithersburg, MD), 36 mL of 5% yeast extract (Sigma-Aldrich, St. Louis, MO) in CMRL 1066, 5.6 mL of 30% glucose (Sigma-Aldrich) in distilled water, 1.4 mL of 5% dog bile in PBS, 20 mM HEPES (Sigma-Aldrich), 10 mM NaHCO3 supplemented with penicillin (100 IU/mL), streptomycin (100 mg/mL), and (ii) the solid phase that is bovine serum coagulated at 76 °C for 20–30 min^42,46^. The medium was changed every 7 days and viability and morphological development of the parasite was observed under an invert microscope (TCM 400, Labomed Inc., CA).

For monophasic culture the protoscoleces were cultivated in Dulbecco’s minimal essential medium (DMEM) (Gibco, Grand Island, NY) containing 10% heat-inactivated FBS (Gibco), 2 mM glutamine (Sigma-Aldrich), penicillin (100 IU/ml), and streptomycin (100 mg/ml) at the bottom of 25 cm^2^ flasks incubated at 37 °C with 5% CO2. The medium was changed every 8 days. The flasks containing the protoscoleces were monitored weekly under an optical microscope to check the growth status of the microcysts^5,42,47,48^.

### Designing and Synthesis of small interfering RNA

We used several online softwares for siRNA design i.e. siDirect (sidirect2.rnai.jp/), siRNA Design (Integrated DNA Technologies, IDT), BLOCK-iT RNAi Designer (www. invitrogen.com/rnai), and RNA wizard (https://www.invivogen.com/sirnawizard). After prediction, all siRNAs were manually re-checked relative to their position, target site, length of siRNA, nucleotide content and specificity of siRNA (off targets), and finally the best was chosen. The siRNA was synthesized commercially by TAG Copenhagen A/S (Copenhagen, Denmark). The sequences of the EgCaM siRNA were as follows: sense 5′UCGUUAAAGUCAAUAACACCC3′-fluorescein and antisense 5′GUGUUAUUGACUUUAACGAAU3′ –fluorescein. Another negative (non-silencing) control siRNA was purchased as an unrelated siRNA (Qiagen, Germany).

### siRNA delivery and phenotype studies

Five sets of experiments were designed for each of three developmental stages of *E. granulosus*: (1) no-treatment group without any RNAi intervention, (2) unrelated/irrelevant Negative Control siRNA (siR-Ctrl) group that was transfected into the parasites using three transfection techniques i.e. soaking, electroporation and electro-soaking, (3) EgCaM-specific siRNA group delivered by soaking, (4) EgCaM-specific siRNA group delivered by electroporation and (5) EgCaM-specific siRNA group delivered by electro-soaking^13,14,21,49^. EgCaM-specific and Negative Control siRNAs were delivered into the parasites of corresponding treatment groups using three transfection techniques. No interventions were made on the parasites in no-treatment groups. For siRNA delivery, in 6-well plates different stages of the parasite with three separate batches of 5000 protoscoleces, 15 microcysts and 15 strobilated worms were treated by soaking, electroporation and electro-soaking in the presence of 50 nM/ml of fluorescent siRNA^50^. For soaking, protoscoleces were cultured in 2.5 ml DMEM supplemented with siRNAs and a lab-made transfection reagent was then added to each well and maintained for 6 hours^51^. For electroporation, protoscoleces were removed from the culture, resuspended in 200 μl of electroporation buffer containing the fluorescently labeled siRNA to give a final concentration of 50 nM, transferred to a 2 mm gap cuvette, and were subjected to time constant protocol (125 V, 20 ms) using Gene Pulser II (Bio-Rad, CA)^13,16,31^. For electro-soaking following electroporation, the parasites were maintained in a culture media containing siRNAs for 6 hours. After treatment, the parasites were rinsed thoroughly in PBS to remove unincorporated siRNA molecules. A fluorescent microscope (HB-10101AF, Nikon, Japan) was used to examine siRNA delivery. The parasites viability, as revealed by flame cell activity, were recorded daily for eight days and any phenotypic changes e.g. motility, transparency and tegumental alterations were examined. In addition, the size of the protoscoleces was measured using a calibrated microscope. The motility of the strobilated worms from each group of target siRNA and controls were measured as the number of body contractions per minute (see the Supplementary Fig. S1).

### Gene expression assay

Total RNA was extracted from different developmental stages of the parasite (protoscoleces, microcysts and three or more proglottids) using a commercial kit (RNeasy Mini Kit, Qiagen, Germany). Furthermore, RNA was quantified by measuring the absorbance ratios at 260 and 280 nm by spectrophotometry (Nano Drop ND-1000, Nano Drop Technologies, Wilmington, DE). cDNA was then synthesized from 100 ng RNA in a total volume of 20 μl using miscript®II Reverse Transcriptase Kit (Qiagen, Germany) according to the manufacturer’s instructions.

Real-time qPCR (RT-qPCR) was carried out in a Rotor-Gene Q System (QIAGEN, Hilden, Germany). The primers specifically designed for EgCaM RT-qPCR were CalF (5′-GAAGGA TAC CGA TAG TGA GGA AGA-3′ and CalR 5′-ATC ATT TCG TCA ACC TCC TCG TC-3′). The primers of the ACTB (encoding β-actin) were ACTBF (5′-ATG GTT GGT ATG GGA CAA AAG G-3′ and ACTBR 5′- TTC GTC ACA ATA CCG TGC TC-3′). Relative quantification of gene expression levels was carried out by using SYBR green PCR Master Mix with 5 µl 2X QuantiNova SYBR Green PCR, 0.4 µM primer, 2 µl 0.5X diluted Template cDNA and 2 µl RNase-free water with a final volume of 10 µl. All samples were run in duplicate and underwent 40 cycles of 95 °C, 2 min for initiation, 94 °C, 5 sec for denaturation, and 60 °C, 10 sec for annealing.

For relative quantification, 2^−ΔΔCT^ method was employed, using β-actin as the reference gene for each sample. Results obtained from the parasites treated with the unrelated Negative Control siRNA were used as calibrators. Negative Control siRNA target gene was unknown, as it is the proprietary information of Qiagen^®^. According to the manufacturer, the unrelated siRNA has been proven to have no significant effect on cell proliferation, viability, or morphology.

### Statistical analysis

Differences between and within groups were assessed for statistical significance using one-way or two-way ANOVA test using GraphPad Prism 7.0 Software (www.graphpad.com). P values of less than 0.05 were considered significant.

## Supplementary information

Supplementary Figure S1

## Data Availability

The data of the present study will be available online.
