# Supplementary material for: Calmodulin-specific small interfering RNA induces consistent expression suppression and morphological changes in Echinococcus granulosus
Source: Sci Rep. 2019 Mar 7;9:3894. doi: 10.1038/s41598-019-40656-w (PMC6406006; doi:10.1038/s41598-019-40656-w)
Supplement: Supplementary file 1 — Supplementary Figure S1 [file 41598_2019_40656_MOESM1_ESM.pdf]

Calmodulin-specific small interfering RNA induces consistent expression suppression and morphological changes in *Echinococcus granulosus*

Seyed Mohammad Mousavi [1], Ali Afgar [2], Mohammad Ali Mohammadi [1], Seifollah Mortezaei [1], Balal Sadeghi [3], Majid Fasihi Harandi [1]*

1. Research Center for Hydatid Disease in Iran, Kerman University of Medical Sciences, Kerman 7616914115, Iran

2. Dept of Parasitology, School of Medicine, Kerman University of Medical Sciences, Kerman 7616914115, Iran.

3. Shahid Bahonar University of Kerman, Faculty of Veterinary Medicine, Department of Food Hygiene and Public Health, Kerman, Iran

* Corresponding author:

ORCID ID: 0000-0003-3257-5389

Tel: +98-34-33257316

Fax: +98-34-332 57478

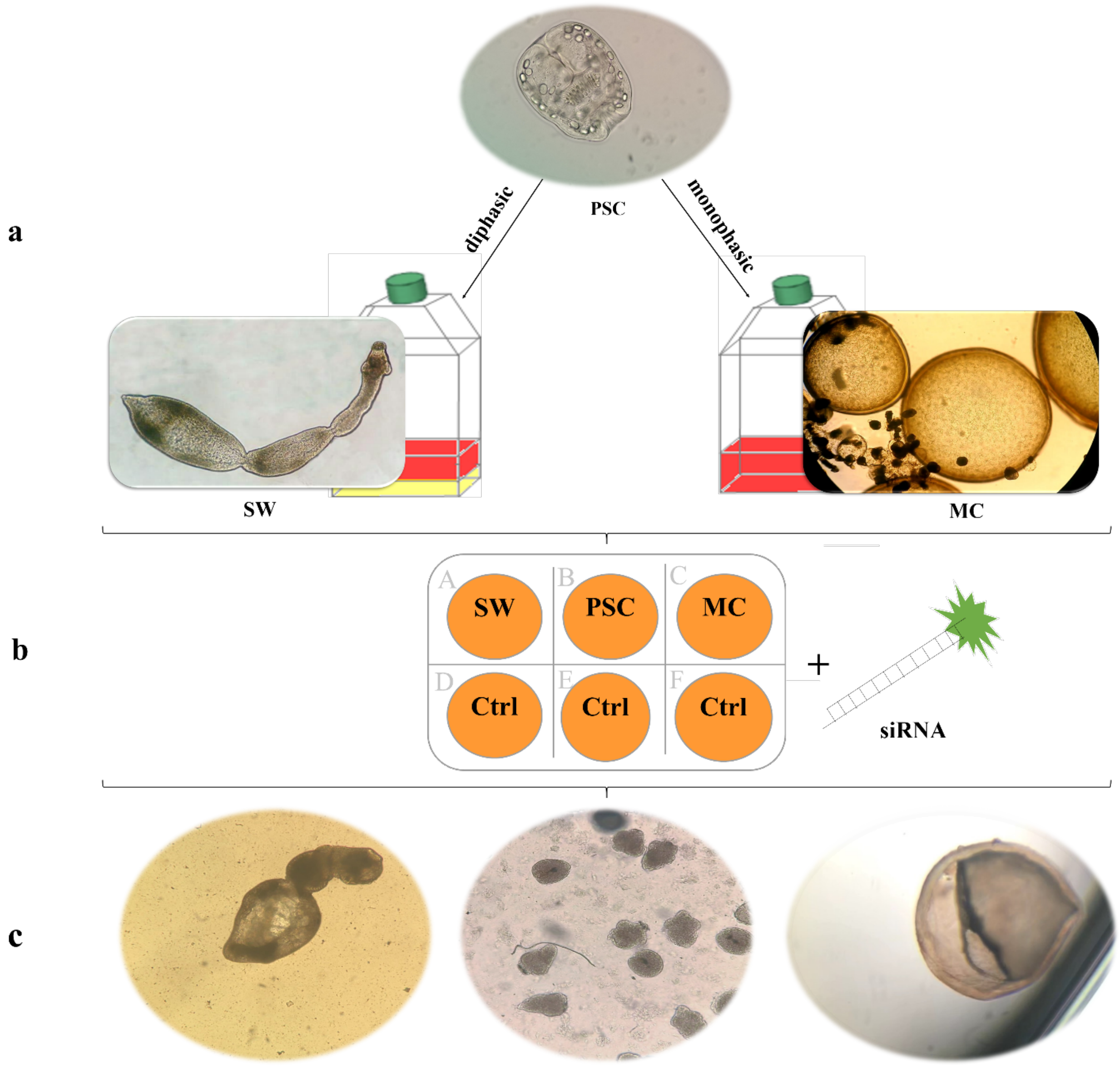

Supplementary Figure S1. Diagrammatic presentation of the expression suppression and morphological anomalies (**c**) induced by Calmodulin-specific small interfering RNA (**b**) on different in vitro developmental stages (**a**) of *Echinococcus granulosus.* Protoscoleces (PSC), Microcysts (MC), strobilated worms (SW), Control (Ctrl).
